# Fostering early adolescent health and planetary well-being through nature: a Delphi study on nature-based literacy

**DOI:** 10.1093/heapro/daaf010

**Published:** 2025-02-26

**Authors:** Michelle Y Barrette, Patti-Jean Naylor, Frederick M E Grouzet, Nevin J Harper

**Affiliations:** Social Dimensions of Health (PhD student), University of Victoria, 3800 Finnerty Road, Victoria, BC V8P 5C2, Canada; School of Exercise Science, Physical and Health Education, University of Victoria, 3800 Finnerty Road, Victoria, BC V8P 5C2, Canada; Department of Psychology, University of Victoria, 3800 Finnerty Road, Victoria, BC V8P 5C2, Canada; School of Exercise Science, Physical and Health Education, University of Victoria, 3800 Finnerty Road, Victoria, BC V8P 5C2, Canada

**Keywords:** nature engagement, Delphi study, literacy, early adolescence, health and well-being

## Abstract

Becoming nature ‘literate’ may promote nature engagement in early adolescence, supporting both health and well-being, while nurturing a sense of environmental stewardship. This study aimed to advance the formative research necessary for the development of a nature-based literacy framework including a set of indicators specific to its measurement during early adolescence. An e-Delphi study design was used with an expert panel comprised of international academics (*n* = 18) and practitioners (*n* = 8). After three rounds, experts agreed key components of nature-based literacy were knowledge, competence, confidence, motivation, experience, connection, and stewardship. The nature-based literacy framework comprehensively represents the intrapersonal factors and related indicators that influence nature engagement, particularly during early adolescence. This will support the development of interventions designed to enhance early adolescent health outcomes, strengthen their connection to nature, and inspire them to value and protect the natural world.

**Contribution to Health Promotion**

- Despite the benefits of nature on health and well-being, early adolescent children are spending less time in nature.
- A comprehensive framework of the intrapersonal-level factors that foster nature engagement during early adolescence is needed.
- This Delphi study explored global expert opinion and reached a consensus on a set of intrapersonal-level factors and indicators that comprise a nature-based literacy framework.
- Becoming nature ‘literate’ may support early adolescent health and well-being while fostering a sense of environmental stewardship.

## BACKGROUND

### Nature engagement is essential for early adolescent health

A growing body of research suggests nature engagement can have a significant impact on human health, especially during early adolescence, a stage of development between the ages of 10 and 14 years (Blum et al. 2014). Empirical studies have identified favourable effects of nature engagement on a variety of health outcomes, including physical, social-emotional, mental, and cognitive health (Chawla et al. 2014, Adams and Savahl 2017, Tillmann et al. 2018, Chawla and Gould 2020, Roberts et al. 2020, Zhang et al. 2020, Barfield et al. 2021, Fyfe-Johnson et al. 2021, Jackson et al. 2021, Vella-Brodrick and Gilowska 2022, Barragan-Jason et al. 2023, Oswald et al. 2023, Puhakka and Hakoköngäs 2024). For instance, the presence of neighbourhood greenspaces is linked with positive physical health outcomes, including enhanced motor skills (Sprague et al. 2022, Stewart et al. 2023), and consistently correlates with greater physical activity levels in early adolescents (Akpınar 2019). Additionally, green schoolyards are associated with improved mental health, emotional well-being, and social development during early adolescence (Chawla et al. 2014, Ly and Vella-Brodrick 2024). Further research shows opportunities for nature engagement during early adolescence not only deepen their connection with nature (Chawla and Gould 2020) but also promote feelings of happiness (Hakoköngäs and Puhakka 2023). Lastly, nature engagement can play an important role in planetary health by fostering a lasting appreciation for nature and developing future environmental stewards (Adams and Savahl 2017, DeVille et al. 2021).

### Philosophy and theories on the human-nature relationship

Engagement with nature lies at the core of the philosophy of human embodiment, which posits that our bodies are the means by which we experience, interact with, and connect to the natural world (Whitehead 2007). Whitehead (2001b) asserts humans are embodied beings, where the mind and body are deeply interconnected, and interacting with nature through physical activities (i.e. hiking, climbing, and gardening) reinforces our connection to the world and enhances our physical, mental, and emotional health. Embodiment philosophy argues that engagement with nature is essential for holistic well-being, as it allows individuals to reconnect with their physicality, ground themselves in the present, and foster a greater sense of belonging to the earth (Durden-Myers et al. 2018).

As well, several theoretical perspectives inform our understanding of the human-nature relationship that aims to explain why we connect with the natural world. Biophilia, first introduced by Wilson (1984), proposed that humans have an innate connection to all forms of life, rooted in our ancestral relationships with the natural world. Kellert and Wilson (1993) elaborated on the biophilia hypothesis, explaining that early humans depended on natural environments for their survival and well-being. Disrupting this connection can lead to negative health consequences. The biophilia hypothesis is supported by numerous studies that highlight the positive effects of interacting with nature on human health (Gullone 2000, Grinde and Patil 2009, Howell et al. 2011, Gaekwad et al. 2022). Furthermore, psychoevolutionary theory, also known as stress reduction theory suggests stress levels are significantly lowered in natural environments because they offer key features essential for human survival, such as water and open spaces (Ulrich et al. 1991). Empirical studies have shown support for Ulrich’s psychoevolutionary theory suggesting non-threatening natural environments lower physiological indicators of stress such as blood pressure, heart rate, and serum cortisol (Yu et al. 2018, Gaekwad et al. 2023). Similarly, attention restoration theory suggests natural settings provide a form of ‘soft fascination’ that gently captures attention and promotes mental rejuvenation, while urban or complex environments require focused attention, which can lead to cognitive fatigue (Kaplan 1995). Several empirical studies support attention restoration theory demonstrating natural environments can facilitate cognitive recovery and improve attention (Berto 2005, Kuo 2015, Stevenson et al. 2018, Moll et al. 2022).

These theories and philosophical insights provide an understanding of the benefits that nature offers for human health, as well as how integrating natural elements can improve our overall quality of life. At the same time, they highlight the inextricable link between human health and planetary health (Ebi et al. 2020). The field of health promotion incorporates planetary health principles calling for the adoption of policies and practices that enhance the well-being of the population by addressing ecological factors that affect its health (WHO 2021). By promoting planetary health through sustainable practices and environmental protection, we not only safeguard human health but also ensure the conservation and sustainability of natural environments for future generations (Howe et al. 2014, Zelenski et al. 2023).

Effective health promotion initiatives that incorporate nature engagement have not only been shown to boost physical and mental health but to encourage environmental stewardship, thus enhancing health for both individuals and the natural environment (Barragan-Jason et al. 2023, Zelenski et al. 2023). In fact, nature engagement during early adolescence helps cultivate a stronger connection to nature, increases ecological knowledge and a sense of ecological citizenship (Frantz and Mayer 2014, Chawla 2018, Kuo et al. 2019, Larson et al. 2019), and is linked to positive environmental attitudes and behaviours in adulthood (Wells and Lekies 2006, Otto and Pensini 2017, Gray and Pigott 2018, Rosa and Collado 2019, Chawla and Gould 2020, DeVille et al. 2021). Engaging with nature provides opportunities to foster a connection and sense of stewardship towards the environment, while also reinforcing the interconnectedness of human health and planetary health.

### Decline in nature engagement during early adolescence

Despite the benefits emphasized in research and theory, there is growing concern about the notable decline in human-nature interactions that tend to begin during early adolescence (Braun and Dierkes 2017, Hughes et al. 2019, Larson et al. 2019, Chawla and Gould 2020, Krettenauer et al. 2020, Soga and Gaston 2022, Almeida et al. 2023). Notable social and physical environmental factors contributing to the decline in nature engagement during early adolescence include the allure of digital devices and social media (Russo and Andreucci 2023), limited access to natural environments (Wall-Reinius et al. 2023), and parental concerns for safety in the outdoors (Truong et al. 2023). While existing interventions such as adventure therapy and wilderness therapy aim to improve early adolescent health and well-being while fostering a stronger connection to the environment (Harper 2017, Kraft and Cornelius-White 2020, Lousen and Andkjær 2024), there is a lack of evidence-based frameworks that guide development of interventions that identify and address the intrapersonal factors affecting early adolescent nature engagement behaviours.

### Health-related literacies that support early adolescent health outcomes

Contemporary transdisciplinary research has begun to identify the intrapersonal factors that contribute to improved health-related outcomes within a more holistic and comprehensive literacy construct (Truman et al. 2020). Studies on empirically defined health-related literacies (i.e. health, food, media, and physical literacy) have revealed a synergistic relationship among their defining components that work together to improve health outcomes among early adolescents (Sum et al. 2018, Vahedi et al. 2018, Fleary and Joseph 2020). Some of these components are shared across types of literacies (Barrette et al. 2022) and comprise intrapersonal factors such as knowledge, motivation, confidence, and competence that enable early adolescents to make decisions and take actions to promote and enhance their health and well-being (Wharf-Higgins et al. 2009, Fleary et al. 2018, Blain et al. 2021, Blaine and Akhurst 2022; Smith et al. 2021). The formative research process of defining other health-related literacies involves several steps beyond an empirical review of the literature (Bröder et al. 2017, Edwards et al. 2017, Fleary 2022). The research process is iterative and requires continual refinement and adjustment to ensure definitions and measures of health-related literacies are accurate, relevant, and effective in improving health outcomes (Carl et al. 2022, Grauduszus et al. 2023).

#### Rationale

Strong evidence exists on environmental factors (social and physical) that drive nature engagement during early adolescence; however, evidence is limited on intrapersonal factors. We need a more holistic and comprehensive understanding of the intrapersonal factors that support early adolescent nature engagement. Nature-based literacy has been suggested, but further research is needed to determine what elements should be incorporated into a holistic comprehensive model. This model should then be tested to support the development and assessment of interventions aimed at fostering early adolescent engagement with nature. In keeping with this more synergistic approach, and recognizing the significance of nature engagement for both human and environmental health, the first step in conceptualizing nature-based literacy has been taken—a proposed definition and a set of intrapersonal factors or components have been identified (Barrette et al. 2022). However, further research is needed to identify and confirm the components and indicators of nature-based literacy.

#### Study aim

Thus, the aim of this Delphi study was to complete the next step in the development of a nature-based literacy framework and indicator set (Barrette et al. 2022) by bringing together an expert panel of academic researchers and professional practitioners to reach a consensus on a comprehensive set of components and associated indicators that define nature-based literacy. Defining these indicators will enable the measurement of nature-based literacy, particularly in early adolescents, and will help evaluate the effectiveness of interventions aimed at enhancing nature-based literacy, and, ultimately, increasing early adolescent engagement with nature. Defining nature-based literacy should support intervention development aimed at improving early adolescent health outcomes, fostering a connection with nature, and motivating them to value and protect the natural world.

## METHODS

The Delphi method is a systematic and iterative approach aimed at achieving consensus among a group of experts to address complex issues (Dalkey and Helmer 1963, Linstone and Turoff 1975, Nasa et al. 2021). The Delphi method has been employed in consensus-seeking studies to identify the components and indicators of health-related literacies (Liao and Lai 2017, Yakar and Karakus 2020, Pavlova et al. 2021). Thus, the method was deemed appropriate for identifying the core components and indicators of nature-based literacy.

The research objectives were to utilize the Delphi method to reach a consensus among experts on (1) a comprehensive set of components that make up nature-based literacy and their indicators, and (2) key age-specific indicators for early adolescents corresponding to each of the core components of nature-based literacy.

### Study design

We designed an exploratory sequential mixed-methods study where qualitative and quantitative data were collected over three Delphi rounds (Keeney et al. 2011, Creswell and Clark 2017, Beiderbeck et al. 2021). We chose an e-Delphi approach that allowed for global recruitment while preserving the anonymity of the panel through the use of technology throughout the study (Shortt et al. 2019). Expert panellists completed a series of iterative surveys in English, which included both open-ended questions and Likert scale rating scales across three rounds. We set an a priori consensus level of 70% based on recommendations indicating that this is a strong cut-off point (Diamond et al. 2014, Slade et al. 2014).

The anonymity of the panel was ensured, and feedback on the surveys remained confidential throughout the Delphi process (Kenney et al. 2011). Ongoing consent was confirmed prior to each Delphi round. Additionally, consent was obtained from third-round panellists who wished to be formally acknowledged in a future publication about the Delphi study. The Delphi process was completed over a period of 13 months, starting with Round 1 data collection from October 2022 to January 2023, then Round 2 data collection from February to April 2023, and ending with Round 3 from June to October 2023.

### Procedure

#### Expert panel recruitment

We aimed to recruit a minimum of 10 panellists; at least five from each of two subgroups (academic researchers and professional practitioners). Inclusion criteria specified at least 5 years of experience in early adolescent development, outdoor education, or nature-based education. In addition, panellists should be practitioners (e.g. adventure program guides) and/or academic researchers employed at a college or university, and they must be proficient in either English or French.

Purposeful searches and reviews of public websites and peer-reviewed journal articles were conducted to compile a list of potential panellists and relevant organizations. Potential panellists were contacted via email using publicly available information. Snowball recruitment occurred as selected panellists shared details about our study with their colleagues. Additionally, third-party recruitment was utilized, with relevant organizations asked to send recruitment letters to their members and/or associates (i.e. CPA Environmental Psychology section; Child and Nature Alliance; Children and Nature Network).

Thirty potential panellists were contacted to introduce our study and 26 returned signed consent forms. Confirmed panellists included 18 academic researchers and 8 practitioners, consisting of 13 males and 13 females and represented Canada, the USA, the UK, Australia, Denmark, Norway, Japan, Portugal, and New Zealand (see Table 1). The study began with 26 panellists in Round 1, but this number dropped to 11 panellists by Round 3.

**Table 1.** Summary of expert panel members

Characteristic	Descriptor	Initial consent to Delphi study (*n* = 26)	Round 1 survey completed (*n* = 19)	Round 2 survey completed (*n* = 15)	Round 3 survey completed (*n* = 11)
Gender	Female	13	10	8	6
	Male	13	9	7	5
Location	Canada	8	6	6	5
	USA	6	6	3	1
	UK	2	2	2	2
	Australia	3	2	1	1
	Denmark	2	1	1	1
	Norway	2	1	1	1
	Japan	1	1	1	0
	Portugal	1	0	0	0
	New Zealand	1	0	0	0
Representation	Academic	18	15	12	10
	Researcher				
	Practitioner	8	4	3	1

### Data collection

#### Delphi survey—Round 1

Open-ended questions designed to stimulate idea generation were used in the first survey. Core components of nature-based literacy identified in the literature by Barrette et al. (2022) were provided to the experts a priori and utilized to frame questions that sought expert opinions about those components and gathered any new insights about new components that could contribute to comprehensive definition of nature-based literacy. The expert panel was invited to respond to the following questions related to identifying the core components and key indicators of nature-based literacy as proposed by Barrette et al. (2022):

- Please list all components (including any of those proposed in Barrette et al. plus any additional components), that you believe are core to a comprehensive definition of nature-based literacy.
- Provide a brief explanation about why you have included the component. If you don’t agree that a component proposed by Barrette et al. (2022) should be included please provide an explanation about this.
- Please identify any key indicators for each component in your list. Indicators are measures that can be used to assess the state or level of the component in an individual.
- Please identify the key indicators that you believe are relevant to early adolescence (ages 12–14) for each component you suggested above.

#### Delphi survey—Round 2

In Round 2, we used SurveyMonkey (www.surveymonkey.com) to generate survey items based on the qualitative data collected during Round 1. This second survey collected both quantitative and qualitative descriptive data. The survey was comprised of 70 items categorized into the following sections:

i. proposed core components of nature-based literacy;
ii. new components;
iii. indicators:
  1. competence indicators (general);
  2. competence indicators—during early adolescence (ages 12–14 years);
  3. confidence indicators;
  4. confidence indicators—during early adolescence (ages 12–14 years);
  5. knowledge indicators;
  6. knowledge indicators—during early adolescence (ages 12–14 years);
  7. motivation indicators;
  8. motivation indicators—during early adolescence (ages 12–14 years);
  9. new component indicators;
iv. general commentary and themes.

Panellists used a 5-point Likert scale to rate their level of agreement on the components that should be included in a comprehensive list of proposed components and indicators of nature-based literacy (1 = strongly disagree; 2 = disagree; 3 = neither agree nor disagree; 4 = agree; 5 = strongly agree). Open-text comment boxes were included to gather additional feedback from panellists that explained their ratings of the items.

#### Delphi survey—Round 3

Fifteen surveys went out to panellists who completed Round 2 and consented to continue with Round 3. Panellists used a 5-point Likert scale to rate their level of agreement and open-text comment boxes were included for additional feedback from panellists that explained their ratings of the items. In response to feedback from the previous round, we revised the third-round survey to present nature-based literacy as a system of interconnected and interacting components. The survey included a summary of the core components, indicators, and early adolescent indicators that achieved consensus in Round 2. Eight survey items that were ‘close to consensus’ during Round 2 were included for reconsideration on the survey for Round 3. These items received 67% agreement, with 10 out of 15 panellists rating them 4 or higher on a 5-point Likert scale (1 = strongly disagree; 2 = disagree; 3 = neither agree nor disagree; 4 = agree; 5 = strongly agree). Featuring the 8 ‘close to consensus’ items, each panellist received a survey designed to display their previous ratings alongside the group’s median rating. We requested panellists to re-rate these items based on the new information gathered from Round 2.

The survey also requested panellists to rename two core components where consensus had been achieved, but were expressed as adverbs or action words (i.e. experiential) rather than concrete nouns (i.e. competence or confidence). We provided panellists with the opportunity to indicate their level of agreement with changing the names to similar words that preserved their meaning while being measurable. Several panellists misunderstood the instructions and did not rate these survey items, leading to their removal from quantitative analysis. However, qualitative data collected on renaming supported changing the new component advocacy to *stewardship* and experiential to *experience.*

### Data analysis

#### Delphi survey—Round 1

Nineteen surveys were completed and analysed during Round 1. Qualitative data were prioritized in the first Delphi survey and were used to create survey items for quantitative analysis in the second Delphi survey (Round 2). The analysis of survey questions utilized the six-step process of thematic analysis, as outlined by Braun and Clarke (2006): (i) familiarizing ourselves with the data, (ii) conducting initial coding, (iii) searching for themes, (iv) refining themes, (v) defining and naming themes, and (vi) analysing and reporting the results. The analysis process began with a deductive approach, where semantic coding and theme development (Braun and Clarke 2006) were guided by a proposed conceptual framework of nature-based literacy (Barrette et al. 2022) and a priori themes/components. After familiarization and initial coding of key ideas, codes were then grouped within the a priori themes/components where they fit.

At the same time, we utilized an inductive approach for the development of new themes based on the content of the data that went beyond the a priori set of themes (Braun and Clarke 2006, Naeem et al. 2023). As with the process of thematic analysis, described above, the data were explored for key ideas and grouped into codes. Themes were then developed, refined, and named from grouped codes. For example, in the development of new component themes, key meaningful quotes or phrases were used for initial coding (e.g. emotional connection with nature, sense of belonging in nature, sense of comfort, solace, and inner peace within natural surroundings) and then those codes were grouped and used to develop a summarizing theme like connection. The inductive approach was also applied to analyse expert general comments that, while not directly addressing specific components of the framework, focused on contextual issues and considerations that should be reflected in the framework and approach to nature-based literacy. These themes were reported on and described as additional themes developed from expert comments. We acknowledge our analysis is shaped by personal experiences, values, and beliefs which may have influenced the research process (Braun and Clarke 2019).

#### Delphi survey—Rounds 2 and 3

Fifteen surveys were completed and analysed during Round 2, and 11 surveys were completed and analysed during Round 3. Quantitative data were prioritized to summarize consensus on the second and third Delphi surveys, while qualitative data were integrated into both surveys to seek explanations on item ratings and to highlight issues of concern or importance. Quantitative data gathered from Likert scale ratings from both surveys were analysed using descriptive statistics (i.e. percentage, median, mean, and mode) to determine the level of consensus on the survey items. The inductive approach to thematic analysis (Braun and Clarke 2006) was also used to analyse for any new comments and issues that may have been raised by the experts regarding the model or overall discussion of nature-based literacy.

## RESULTS

Results of this Delphi study are presented according to each round. Key descriptives for the expert panel are provided in Table 1.

### Delphi survey—Round 1

Out of the 26 experts who agreed to participate in the study, 19 completed the survey in the first round. Table 2 summarizes key ideas that were coded and grouped into deductive and inductive themes related to components of the nature-based literacy framework.

**Table 2.** Coding of key themes for components, indicators, and early adolescent indicators (Round 1)

Theme	Key ideas/codes
Components	
Competence	Practical skills related to interacting with and navigating through nature. Physical, social, cognitive, and psychological skills necessary for effective engagement in nature. Competence varies depending on age, gender, ability levels. Competence is contextual and influenced by cultural perspectives.
Confidence	Self-efficacy resulting from knowledge and competence. Self-assurance and comfort in engaging with nature, including facing challenges and uncertainties.
Motivation	Cognitive and emotional factors that drive engagement with the natural environment. Recognizing various factors influencing motivation for engage with nature, including intrinsic interests, external influences, cultural values, and personal goals.
Knowledge	Understanding of the interconnectedness and functioning of natural systems, as well as one’s place within nature. Understanding ecological concepts and biodiversity. Acknowledging diverse ways of knowing based on cultural, personal, and historical contexts.
Connection^a^	Emotional connection with nature. Sense of belonging in nature. Sense of comfort, solace, and inner peace within natural surroundings.
Experiential^a^	Learning through hands-on activities and observation. Sensory engagement promoting mindfulness and presence in nature. Observation and appreciation of the environment.
Advocacy^a^	Caring for nature. Direct actions such as environmental conservation and stewardship to support sustainability-oriented initiatives.
Place^a^	Bidirectional relationship between humans and nature. Sense of place, attachment, and identity with natural environments. Cultural, spiritual, and ancestral significance of nature. Cultural and historical implications on one’s relationship with nature. Accessible and secluded spaces within nature.
Indicators	
Competence	Outdoor survival skills such as building a campfire, setting up a tent. Outdoor safety skills including navigation, map reading, and orienteering. Outdoor recreational activities such as paddling a canoe, rock climbing, and bird identification. Accurately predict outcomes and consequences of actions in natural environments. Social and collaboration skills during outdoor activities.
Confidence	Sense of comfort and ease within natural environments. Willingness to take risks and face challenges outdoors. Adapting to unforeseen circumstances and setbacks in outdoor experiences.
Motivation	Valuing spending time in nature. Seeking nature for comfort and revitalization. Persistence in outdoor activities despite challenges. Intrinsic motivation driven by personal interests, curiosity, and enjoyment of nature. Extrinsic motivation influenced by social factors, rewards, or incentives for participating in outdoor activities.
Knowledge	Ability to recognize and identify elements of the environment (i.e. flora and fauna). Understanding ecological processes. Understanding human impact on local and global environmental issues. Understanding cultural and natural knowledge about specific places or landscapes. Understanding gear and equipment required for outdoor activities. Understanding environmental conservation principles and practices. Understanding benefits of nature for personal well-being and community health.
Connection	Special attachment to plants or animals in nature. Engagement in activities to care for and nurture nature. Recognition of the importance of conservation and environmental stewardship. Engagement in pro-environmental behaviors and advocacy for sustainable living practices. Measures on connectedness to nature scale (CNS). Feelings of awe, wonder, and reverence for the natural world.
Experiential	Direct and indirect experiences with nature. Quantified nature experiences by type, levels of immersion, duration, and frequency.
Advocacy	Involvement in environmental initiatives. Engagement in pro-environmental behaviours and sustainable living practices.
Place	Use of natural spaces for personal activities and socializing. Measurement of sense of place, place attachment, and emotional connection to specific natural places.
Early adolescent indicators	
Competence	Basic survival skills such as choice of appropriate outdoor clothing, assessing and managing risks in outdoor activities. Basic safety skills using navigational tools. Basic outdoor recreational activities such as use of guidebooks to identify flora and fauna; participate in outdoor activities such as swimming, hiking, and canoeing.
Confidence	Comfort in outdoor settings and nature-based activities. Belief in one’s ability to engage in outdoor activities.
Motivation	Desire and enthusiasm for spending time outdoors. Engagement in nature-based activities. Willingness to face challenges and try new experiences in nature.
Knowledge	Ability to identify flora and fauna species. Ability to identify local plants with edible or medicinal properties. Awareness of the benefits and risks associated with nature. Understanding of ecological concepts such as biodiversity and ecosystem functioning. Understand human impacts on environment and conservation issues. Familiarity with outdoor safety practices such as hiking with a buddy, carrying water, and informing someone of their whereabouts.
Connection	Care for plants, animals, and natural environments. Measures of belonging and identity with natural environment.
Experiential	Reflective statements, narratives, and photos.
Advocacy	None indicated in questionnaire.
Place	Attachment to specific natural areas as personal retreats or hideaways. Artistic representation of special natural areas

^a^New components identified by expert panel after Round 1.

#### Additional themes developed from expert comments

**Figure 1.**
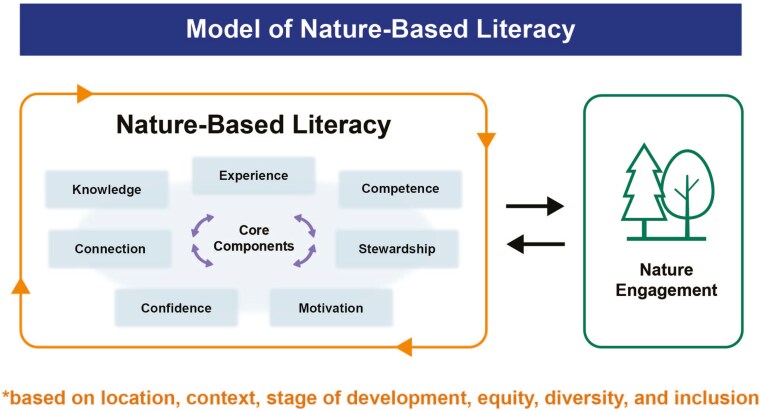
Model of nature-based literacy.

Inductive thematic analysis of the expert panel’s general comments provided valuable insights into nature-based literacy. Three new themes of importance were developed from the data and are described below. Several participant quotes have been included in support of these new themes. Important expert feedback that could not be themed is also described, as it relates to considerations for component definitions. These additional themes and definitions were used to refine the model of nature-based literacy (see Fig. 1).

#### Equity, diversity, and inclusion

A theme of equity, diversity, and inclusion developed from expert recommendations that nature-based literacy be framed to reflect the inherent differences among human populations and their living environments.

*Factors such as culture, location (e.g., urban, rural), access, and other systemic & historic contexts affecting people’s relationship with nature likely shape the contours of an individual’s nature-based literacy.*

In terms of measures of nature-based literacy, experts suggested indicators need to consider a variety of abilities aimed at fostering nature engagement for everyone. Experts also noted equity and accessibility as critical factors that impact opportunities for nature engagement, necessitating the removal of barriers to ensure equal nature experiences for everyone.

*Can navigate different kinds of terrain in age- and ability-appropriate ways (wouldn’t expect a child using a walker to navigate in the same way).*

Furthermore, gendered experiences were identified as factors that may influence the development of competence and should be considered when assessing this component. While knowledge was noted to be cultural, contextual, and place-based, and its indicators should reflect diverse ways of knowing and understanding.

*Can identify the typical uses of certain plants and animals in indigenous and non-indigenous traditional and contemporary practice – for medicine, for spiritual practice, for food.*

*Knowledge is important to develop a lifelong sense of environmental stewardship, but also to feel comfortable going into a diversity of natural spaces.*

#### Risk tolerance and management

A theme of risk tolerance and management developed throughout the data and was characterized as an intrinsic quality that influences nature engagement. Experts suggest the ability to discern, adapt, and overcome challenges is vital for managing risk and crucial for developing nature-based literacy. Experts also identified the willingness to step outside of comfort zones is essential for developing competence, confidence, and motivation in nature.

*Understands risk vs. danger and can use that knowledge to make a good decision.*

*Motor, survival, and risk management skills are essential components of nature literacy.*

#### Interconnected and embedded

A theme of interconnected and embedded was also identified in the data addressing the model of nature-based literacy. Expert feedback emphasized integration of components, indicating that the qualities associated with the proposed components of nature-based literacy influence each other’s development. For instance, confidence both influences and is influenced by competence, while knowledge contributes to confidence, which in turn can enhance knowledge further.

*We might be more inclined to recognise motivation as influenced by factors such as knowledge (through the impact on attitudes), competence and confidence.*

*I believe confidence and competence are intimately related.*

*Conceptually, I don’t see confidence as being it’s* [*sic*] *own component, but rather a requirement in order to achieve competence.*

*No competences without knowledge*

As such, the components in the model should illustrate interconnection and embeddedness by overlapping and nesting them within one another, highlighting the multi-directional nature of the interactions among components.

#### Definitions

While not themed, some important expert feedback highlighted a number of considerations for defining the proposed components of nature-based literacy (Barrette et al. 2022). Some comments emphasized that competence in nature is distinct from being physically literate outdoors and should not be confused with the concept of physical literacy which has different outcomes compared to nature-based literacy.

*Competence in the outdoors/nature goes well beyond physical literacies/the ability to gain locomotor patterns in the outdoors-the term has been used to describe technical skills, interpersonal skills, leadership skills, and self-care skills as well as the ability to move through/on terrain and water.*

Some experts also noted the description of the knowledge component was confined to a taxonomic understanding. They proposed the definition of knowledge should encompass forms of self-knowledge (such as embodied, kinesthetic, and emotional sensing), and factual, conceptual, and procedural knowledge.

*Both tacit and explicit knowledge about the natural world is essential for building and maintaining a relationship with and understanding of nature.*

*This might include things like natural history and identification knowledge (especially local), ecological knowledge, knowledge of where/how to access natural areas, knowledge of benefits of nature.*

Further feedback focused on the motivation component, suggesting the definition should outline how it manifests in the affective, cognitive, and behavioural domains, and could also reference the self-determination continuum as described by Ryan and Deci (2000).

*I would recommend to add Self-Determination Theory’s spectrum from a-motivation to intrinsic motivation to describe the simplex-structure of motivation.*

### Delphi survey—Round 2

#### Rating of survey items

During Round 2, 19 surveys were emailed to consenting Delphi panellists and 15 surveys were returned. Five component, 7 indicator, and 9 early adolescent indicator survey items reached consensus^1^ during Round 2. Eight close to consensus^2^ survey items were kept and used for reconsideration in Round 3. Table 3 presents descriptive statistics for consensus and ‘close to consensus’ survey items including the percentage of panellists who rated survey items 4 or 5 (out of 5), along with the range, mean, median, and mode ratings. No new information was gleaned from qualitative data this round.

**Table 3.** Descriptive summary of component, indicator, and early adolescent indicator survey items with consensus and close to consensus (Round 2)

Survey item number	Items	Tally number of survey ratings of 4 + (out of 15)	Percentage	Mean	Median	Mode	Range
Components							
Consensus component items^a^							
1	Competence	11	73.33	4.13	4	4	2
3	Knowledge	15	100.00	4.53	5	5	1
6	Connection	14	93.33	4.73	5	5	2
7	Experiential	13	86.67	4.20	4	4	3
8	Stewardship	11	73.33	4.07	4	5	3
Close to consensus component items^b^							
2	Confidence	10	66.67	3.93	4	4	2
4	Motivation	10	66.67	3.87	4	4	3
Indicators							
Consensus indicator items^a^							
15	Competence measures of subcomponents (social, physical, and cognitive skills)	12	80.00	4.00	4	4	3
21	Confidence measures as one’s belief in their ability to engage in nature-based activities	12	80.00	4.07	4	4	2
30	Procedural knowledge measures	11	73.33	3.87	4	4	3
31	Metacognitive knowledge measures	11	73.33	3.80	4	4	3
47	Connection measures (inclusion of self- or nature-relatedness scales)	12	80.00	4.07	4	4	2
48	Connection measures (affective, cognitive, and behavioural components)	11	73.33	4.00	4	4	2
49	Connection measures (to what degree people feel a part of, or belonging with nature)	11	73.33	4.07	4	5	3
Indicator survey items close to consensus^b^							
22	Confidence measure (‘I am confident /I could perform x behaviour’)	10	66.67	3.67	4	4	3
28	Factual knowledge measures	10	66.67	3.67	4	4	3
29	Conceptual knowledge measures	10	66.67	3.93	4	4, 5	3
38	Affective domain measures	10	66.67	4.00	4	5	4
50	Connection measures (sense of place; place attachment)	10	66.67	4.07	4	5	3
Early adolescent indicators							
Consensus early adolescent indicator items^a^							
17	Competence (age-specific measures of basic safety skills)	11	73.33	3.73	4	4	3
18	Competence (age-specific measures of basic survival skills)	12	80.00	3.93	4	4	2
19	Competence (age-specific measures of recreational skills)	12	80.00	4.00	4	4	2
25	Confidence (age-specific measures of level of comfort in nature)	11	73.33	3.73	4	4	3
33	Knowledge (age-specific measures of factual knowledge)	11	73.33	3.87	4	4	3
34	Knowledge (age-specific measures of conceptual knowledge)	11	73.33	3.87	4	4	3
35	Knowledge (age-specific measures of procedural knowledge)	11	73.33	3.67	4	4	3
36	Knowledge (age-specific measures of metacognitive knowledge)	11	73.33	3.80	4	4	3
51	Connection (age-specific measures of experience nurturing plants and wildlife)	11	73.33	3.80	4	4	3
Early adolescent indicator survey items close to consensus^b^							
53	Connection (age-specific measures of Inclusion of Self in Nature scale)	10	66.67	3.93	4	4	2

^a^Meeting the a priori threshold of 70% of survey respondents rating this item 4+.

^b^67% of survey respondents rated this item 4+.

### Delphi survey—Round 3

#### Rating of ‘close to consensus’ survey items

During Round 3, a total of 11 surveys were completed and returned by the Delphi panel. All 8 ‘close to consensus’ survey items, from Round 2, reached consensus in Round 3. Table 4 presents descriptive statistics for these 8 items, including the percentage of panellists who rated survey items 4 or 5 (out of 5), along with the range, mean, median, and mode ratings. In total, we compiled a list of 7 components, 12 indicators, and 10 early adolescent indicators that reached consensus after Rounds 2 and 3 (see Tables 3 and 4). No new information was gleaned from qualitative data this round.

**Table 4.** Descriptive summary of ‘close to consensus’ component, indicator, and early adolescent indicator survey items now with consensus (Round 3)

Survey item number	Items	Tally number of survey ratings of 4 + (out of 15)	Percentage	Mean	Median	Mode	Range
Components							
Consensus component items^a^							
2	Confidence	11	100	4.27	4	4	1
4	Motivation	10	90.91	4.18	4	4	2
Indicators							
Consensus indicator items^a^							
22	Confidence measure (‘I am confident /I could perform x behaviour’)	10	90.91	4.00	4	4	2
28	Factual knowledge measures	9	81.82	4.09	4	4	2
29	Conceptual knowledge measures	11	100	4.36	4	4	1
38	Affective domain measures	10	90.91	4.36	4	4, 5	2
50	Connection measures (sense of place; place attachment)	10	90.91	4.45	5	5	2
Early adolescent indicators							
Consensus early adolescent indicator items^a^							
53	Connection (age-specific measures of Inclusion of Self in Nature scale)	10	90.91	4.18	4	4	2

^a^Meeting the a priori threshold of 70% of survey respondents rating this item 4+.

## DISCUSSION

Nature-based literacy is a new concept first proposed by Barrette et al. (2022). This study advanced the conceptualization and operationalization of nature-based literacy. Academic and professional practitioners in the fields of early adolescent development, outdoor education, adventure-based and nature-based education were instrumental in further refining a definition of nature-based literacy and the findings introduced novel components for inclusion into a conceptual model of nature-based literacy. After three rounds, experts reached a consensus on a set of components, indicators, and early adolescent indicators that comprise and inform a nature-based literacy framework. After further development of nature-based literacy, our study identified a set of intrapersonal factors that may influence early adolescent engagement with nature.

### Intrapersonal factors that promote nature engagement in early adolescence

The expert panel agreed on the following intrapersonal factors that promote nature engagement during early adolescence: knowledge, competence, confidence, and motivation.

Experts also agreed on the inclusion of three new components to be included in the definition of nature-based literacy: experience, connection, and stewardship. Some of these intrapersonal factors (i.e. confidence, competence, motivation, and knowledge) have also emerged in other health-related literacies such as health literacy (Nutbeam and Kickbusch 1998, Ratzan and Parker 2000, Zarcadoolas et al. 2005, Baker 2006, Parnell 2015), physical literacy (Whitehead 2001b, Almond 2013, Tremblay et al. 2018), nutrition literacy (Zoellner *et al*. 2009, Cullen et al. 2015, Velardo 2015), and ecological literacy (Orr 1992, Berkowitz et al. 2005, Balgopal and Wallace 2009, Mitchell and Mueller 2011).

### Defining nature-based literacy

The results of our study suggest nature-based literacy is comprised of a set of seven components: (i) knowledge, (ii) competence, (iii) confidence, (iv) motivation, (v) experience, (vi) connection, and (vii) stewardship (see Fig. 1).

The model of nature-based literacy that emerged from expert feedback illustrates an overlapping or interwoven structure, highlighting the multi-directional interactions that occur among the components in the model. Specifically, the model emphasizes the interconnectedness of competence, confidence, and motivation. This model aligns with psychosocial literature that examines the dynamic interaction between confidence and competence and how these qualities subsequently influence motivation (Bandura and Schunk 1981, Ryan and Deci 2000). The model also highlights that the development of nature-based literacy may occur at different times, may vary across populations and geographical regions, and may be limited by one’s access to nature. This is consistent with literature that explored how diverse abilities impacted engagement with nature (Guest et al. 2023, Wall-Reinius et al. 2023) while recognizing access to nature was not equitable (Anderson et al. 2023, Harrison et al. 2023, Teeuwen et al. 2023, Ghanem and Edirisinghe 2024). Finally, this study’s findings align with literature that suggested knowledge about nature is influenced by cultural, contextual, and place-based factors, and should consider diverse perspectives (Henri *et al.* 2021, Boyd et al. 2024).

### Nature-based literacy for early adolescent and planetary health

This study’s findings align with nature theories that suggest nature engagement is intimately linked with human health, and support calls for promoting human health and well-being through nature engagement health (Ulrich et al. 1991, Kellert and Wilson 1993, Kaplan 1995, Richardson et al. 2016, Phillips et al. 2023). Identified indicators of nature-based literacy contribute to the health and well-being of early adolescents by fostering the development of outdoor knowledge and skills, which in turn support their engagement in nature-based activities. The findings also support a call to action on planetary health initiatives that aim to understand and address the intrapersonal factors (i.e. attitudes, values, and behaviours) driving ongoing environmental destruction imposed by human activity, and promoting education for lifelong health and well-being while nurturing environmental stewards from an early age (Hansen-Ketchum and Halpenny 2011, Prescott et al. 2022, Zelenski et al. 2023). These findings align with existing literature indicating early adolescents who engage in nature tend to feel a stronger connection to nature and are more inclined to take action to care for it, extending into adulthood (Chawla and Gould 2020, Anderson and Krettenauer 2021).

## STRENGTHS AND LIMITATIONS

The study had a number of strengths. Expert consensus is a recommended approach when ideas and concepts are emerging (Nasa et al. 2021) and the Delphi method is well-known and appropriate (Dalkey and Helmer 1963, Linstone and Turoff 1975). The inclusion of both practitioners and academics enhanced the depth of the opinions. Expertise in their respective fields contributed to a panel offering rich and detailed information that supported our study’s objectives of identifying an agreed-upon comprehensive list of components and indicators that comprise nature-based literacy. While consensus was reached on components and indicators of nature-based literacy, the findings should be considered in light of the limitations. Firstly, reaching a consensus does not mean we have found the correct answer to our questions. As well, we experienced a notable drop in participation over the course of a year-long study. A systematic bias may have been introduced into the ratings based on who remained. Accordingly, the model and definitions may require further review, validation, and even revision to bring it closer to the best international practices. Some ethical considerations were also noted during the study including the labour-intensive nature of completing three Delphi rounds. Finally, our study lost mostly practitioners, as panellist numbers dropped over the three rounds. This loss of the practitioner perspective may have influenced the final results.

## CONCLUSION

Engagement with nature tends to decline during early adolescence which carries implications for both human and planetary health. A nature-based literacy framework has been conceptualized that outlines the intrapersonal factors that promote nature engagement during early adolescence. This Delphi study leveraged expert opinion to identify a set of components and indicators of nature-based literacy with the ultimate aim of supporting nature engagement during early adolescence. This study completes the next phase in the formative process of defining a nature-based literacy framework (Barrette et al. 2022).

Defining the components and indicators of nature-based literacy, particularly in early adolescence, will help researchers evaluate the effectiveness of interventions aimed at enhancing nature-based literacy, and, ultimately, enhance early adolescent health and planetary health through engagement with nature. This framework may support health, education, and recreation professionals to foster connection between early adolescents and the outdoors but it needs further validation overall and with youth specifically. Future research should involve the construction of measures, population surveys to test the framework and intervention development and evaluation to further test the constructs and establish the outcomes of intervention.

## RECOMMENDATIONS

This nature-based literacy framework may support health, environmental education, and outdoor recreation professionals to foster a connection between early adolescents and nature, but it needs further validation overall, specifically with early adolescents. The next steps should include finalizing the framework based on feedback from early adolescents with nature engagement experiences. Indicators of nature-based literacy should then be tested with a group of early adolescents to determine their predictive value, followed by intervention development and evaluation to further test the constructs and establish the outcomes of intervention. Intervention programming aimed at developing the intrapersonal factors of nature-based literacy will enhance health and well-being while nurturing a connection and sense of stewardship with nature.

## Data Availability

The datasets used and/or analysed during the current study are available from the corresponding author upon reasonable request.
